# Effects of Bio-Based PP-g-IA Compatibilizer on Polypropylene Composites Incorporating Unmodified and Hydrophobically Modified Lignocellulose

**DOI:** 10.3390/polym18151826

**Published:** 2026-07-25

**Authors:** Seong Jae Cho, Hyeon Soo Kwon, Tae Hyun Kim, Dong-Hyun Kim, Jung-Soo Kim

**Affiliations:** 1 User Convenience Technology R&D Department, Korea Institute of Industrial Technology (KITECH), Ansan 15588, Gyeonggi do, Republic of Korea; zkxm5698@kitech.re.kr (S.J.C.); dhkim@kitech.re.kr (D.-H.K.); 2 Department of Materials Science and Chemical Engineering, Hanyang University, Ansan 15588, Gyeonggi do, Republic of Korea; hitaehyun@hanyang.ac.kr; 3 Department of Carbon Innovation and Energy Convergence, Kangwon University, Chuncheon 24341, Gangwon do, Republic of Korea; khs77235@kitech.re.kr; 4 Major in Advanced Materials and Semiconductor Engineering, School of Semiconductor Convergence Engineering, Hanyang University, Ansan 15588, Gyeonggi do, Republic of Korea

**Keywords:** bio-based compatibilizer, itaconic acid, aspect ratio, processability, interfacial adhesion

## Abstract

In this study, a bio-based compatibilizer, itaconic acid-grafted polypropylene (PP-g-IA; IP), was synthesized via melt grafting as a sustainable alternative to the conventional petroleum-based maleic anhydride-grafted polypropylene (PP-g-MAH; MP). Successful grafting was confirmed by Fourier-Transform Infrared (FT-IR) spectroscopy, and the efficacy of the synthesized compatibilizer was systematically evaluated by applying it to composites incorporating both unmodified lignocellulose (LC) and hydrophobically modified LC (mLC). Mechanical evaluation revealed that the PP-g-IA-compatibilized LC composite (PPLCIP) achieved the highest tensile strength of 23.5 MPa among all samples. This result is attributed to a synergistic effect: the PP-g-IA compatibilizer significantly improved the interfacial adhesion issue, thereby allowing the intrinsically higher aspect ratio of unmodified LC fibers (3.0 ± 2.1) to serve as the dominant reinforcement factor. While mLC surface modification improved composite performance over unmodified LC even without a compatibilizer, the introduction of a compatibilizer shifted fiber aspect ratio to the dominant factor, resulting in LC-based composites outperforming their mLC-based counterparts. Furthermore, PP-g-IA maintained mechanical performance comparable to that of the conventional PP-g-MAH while simultaneously achieving enhanced melt flowability. These results demonstrate that the bio-based PP-g-IA is an effective and sustainable compatibilization strategy that satisfies both mechanical performance and processability requirements.

## 1. Introduction

Polymer composites are extensively studied materials designed to overcome the limitations of conventional materials and achieve superior properties tailored for specific purposes [1]. In particular, thermoplastic composites based on polyolefins, such as polypropylene (PP), play a crucial role in the plastics industry due to their excellent processability, light weight, and cost-effectiveness [2,3,4]. Consequently, they are widely recognized as key materials for light weighting and practical applications in industries such as automotive, sustainable building materials, and eco-friendly packaging [5,6,7]. However, with increasing societal demands for sustainable materials due to environmental concerns associated with petroleum-based polymers and limited resources, research into composites incorporating eco-friendly natural fibers is actively progressing [8,9,10,11,12].

In this context, lignocellulose has garnered significant attention as an excellent alternative. Lignocellulose is an abundant natural polymer found in the plant kingdom, offering advantages such as renewability, biodegradability, and low cost [13,14,15]. Furthermore, it possesses the benefit of not producing toxic by-products, unlike inorganic materials such as glass fibers, carbon fibers, talc, clay, and synthetic fibers [16,17,18,19]. Thanks to these advantages, lignocellulose holds great potential as a reinforcing agent or fiber in polymer composites. In particular, the inherent high stiffness and aspect ratio of natural fibers enable effective stress transfer [1,20,21,22].

However, fundamental limitations exist when applying lignocellulose to polymer matrices. Most general-purpose polymers, particularly polyolefin types such as polypropylene (PP), are non-polar and exhibit hydrophobic characteristics [23,24]. In contrast, lignocellulose, due to the numerous hydroxyl (-OH) groups within the structure of its main components, cellulose and hemicellulose, possesses a highly polar and hydrophilic surface [25,26]. This difference in chemical properties leads to incompatibility between the polymer matrix and the lignocellulose fibers [27,28,29,30]. Consequently, the low compatibility between hydrophilic lignocellulose fibers and the hydrophobic polymer matrix weakens interfacial adhesion and causes fiber aggregation [29,30,31]. This prevents the effective realization of the fibers’ properties, thereby becoming a primary cause of reduced mechanical properties in composites [27,32,33,34].

Recently, various natural-based additives have been explored for polypropylene composites, ranging from natural antioxidants, such as vitamin E for melt stabilization [35], to natural fiber reinforcements, such as lignocellulose. However, when natural fibers such as lignocellulose are used as structural reinforcements, a critical limitation arises: their abundant hydroxyl groups impart strong hydrophilicity, leading to moisture absorption, fiber swelling, and consequent dimensional instability of the composites. In contrast, chemically modified or synthetic additives can circumvent this moisture-related issue by reducing the hydrophilic character of the fiber surface, thereby enhancing compatibility with the hydrophobic PP matrix.

To address these interfacial issues, two main approaches have been intensively investigated in previous studies. The first involves chemically modifying the surface of lignocellulose fibers to alter their surface properties to be more hydrophobic. Surface treatments such as acetylation, esterification, or the use of silane coupling agents deactivate the hydroxyl groups on the fiber surface, improving wettability with the polymer matrix. Numerous prior studies have demonstrated the effectiveness of these methods in enhancing the mechanical properties of composites [36,37,38].

The second approach involves introducing a compatibilizer. A compatibilizer is an amphiphilic substance that possesses both non-polar, polymer-friendly parts and polar, natural fiber-friendly parts within a single molecule, acting as a bridge at the interface of the two phases. Especially in polypropylene-based composites, maleic anhydride-grafted polypropylene (PP-g-MAH) has been the most representative compatibilizer, proven to improve the interfacial adhesion between the fiber and the matrix [39,40,41,42].

While both natural fiber surface modification and the introduction of compatibilizers like PP-g-MAH have demonstrated their efficacy, a shift in the industrial paradigm towards sustainability and eco-friendliness has highlighted the limitations of existing strategies. Particularly, the most widely used PP-g-MAH inherently possesses a fundamental limitation: it is a petrochemical product reliant on fossil fuels, making it difficult to achieve overall eco-friendliness in composites [43,44].

In this study, we aimed to propose a sustainable compatibilization strategy as an alternative to the conventional petroleum-based approach. To this end, we focused on itaconic acid (IA), a representative bio-based chemical derived from the fermentation of biomass, as a sustainable grafting monomer to replace petroleum-based MAH. IA possesses a reactive carbon–carbon double bond suitable for melt grafting onto the PP backbone, along with two carboxylic acid groups capable of forming strong interfacial interactions with the hydroxyl groups of LC. We successfully grafted this eco-friendly itaconic acid onto polypropylene chains, synthesizing a bio-based compatibilizer, PP-g-IA.

Most previous studies have treated fiber surface modification and compatibilizer introduction as separate strategies [36,37,38,39,40]. We hypothesized that the synthesized bio-based PP-g-IA would effectively resolve the severe polarity mismatch between the PP matrix and unmodified LC through robust hydrogen bonding. Furthermore, we postulated that once this critical interfacial barrier is chemically overcome, the inherently higher aspect ratio of unmodified LC would emerge as the dominant physical reinforcing factor, ultimately leading to superior mechanical performance compared to the hydrophobically modified mLC system. To test this hypothesis, this study systematically investigates the interplay and competitive effects between chemical compatibility (via surface modification) and physical reinforcement (via aspect ratio) when applying the bio-based compatibilizer to both unmodified and hydrophobically modified LC systems. Through a comprehensive analysis of melt flow index (MFI), dynamic rheological properties, scanning electron microscopy (SEM), and tensile properties, the microscopic interfacial interactions are directly correlated with the macroscopic mechanical performance of the biocomposites.

## 2. Materials and Methods

### 2.1. Materials

The polypropylene (PP, product name: YUHWA POLYPRO SB9230) used in this study was supplied by Korea Petrochemical Ind. Co., Ltd., Seoul, Republic of Korea. SB9230 is a high-impact block copolymer with a melt flow index of 30 g/10 min (230 °C/2.16 kg), a density of 0.91 g/cm^3^, and a melting point of 167 °C. Itaconic acid (IA, 99% purity) for graft copolymerization and Di-tert-butyl peroxide (DTBP, 98% purity) used as an initiator were purchased from Sigma Aldrich, St. Louis, MO, USA. Additionally, commercial maleic anhydride-grafted polypropylene (PP-g-MAH, MP600PP) was purchased from Chemko S. C. Corp Co., Ltd., Cheonan, Republic of Korea, for comparison with the synthesized compatibilizer.

The lignocellulose (LC, product name: SSEIF-L) used as fiber was supplied by Lignum Co., Daejeon, Republic of Korea. SSEIF-L is a lignin/cellulose-based functional biofiller (CAS No. 9005-53-2) with a density of 1.2 g/cm^3^, moisture content ≤ 3%, median particle size Dv50 = 14.7 μm, pH 6–8, and thermal decomposition temperature > 250 °C. Hydrophobically modified lignocellulose (mLC) was supplied by the Korea Photonics Technology Institute (KOPTI, Gwangju, Republic of Korea). The mLC was hydrophobically surface-treated using a plasma-enhanced chemical vapor deposition (PECVD) [45]. Xylene and acetone, used as washing solvents to remove unreacted materials, were also purchased from Sigma Aldrich. All reagents used were utilized without further purification.

### 2.2. Preparation of PP-g-IA Compatibilizer

The PP-g-IA compatibilizer was prepared via the melt grafting method. After preheating a melt blender (Model: Plasti-Corder 680135, Brabender, Duisburg, Germany) to 190 °C, PP (47.5 g, 95 wt%), IA (2.5 g, 5 wt%), and the initiator DTBP (0.5 g, 1 phr) were added into the mixing chamber. The mixture was then melt-blended at a rotation speed of 60 rpm for 7 min to proceed with the grafting reaction. The obtained product was pulverized and completely dissolved in xylene at 140 °C. Subsequently, this solution was precipitated by pouring into an excess amount of acetone. The resulting precipitate was then filtered using a vacuum aspirator, and finally dried in a vacuum oven at 60 °C for 24 h to obtain the purified PP-g-IA compatibilizer.

**Scheme 1:**
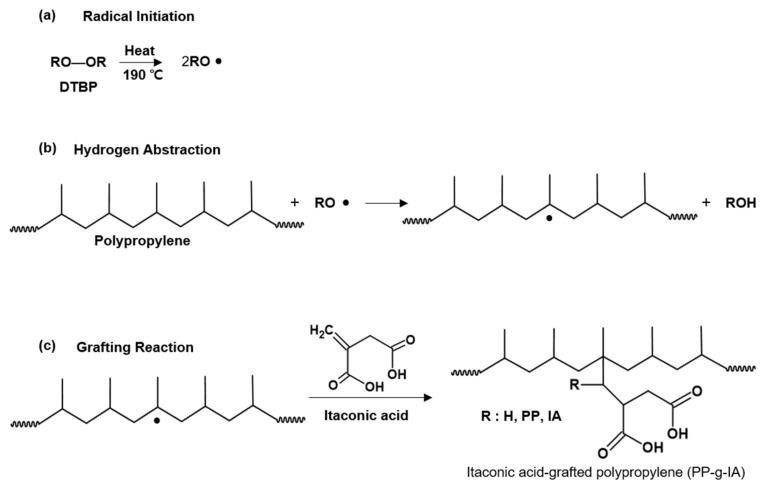
Chemical Reaction Mechanism for Grafting IA onto PP Backbone.

The initial step of the melt grafting process involves the generation of macroradicals on the PP backbone. As depicted in Scheme 1, the peroxide initiator abstracts a hydrogen atom primarily from the tertiary carbon of the PP chain, providing reactive sites for the subsequent grafting of IA monomers.

### 2.3. Preparation of PP/Lignocellulose Composites

Prior to extrusion, the lignocellulose fibers and PP matrix were dried in a convection oven at 60 °C for 24 h to remove moisture. The PP matrix, lignocellulose fibers, and compatibilizers were dry-blended according to the formulations listed in Table 1. The blend was then melt-extruded using a twin-screw extruder (BA19, Bautek, Hwaseong, Republic of Korea). The temperature profile of the extruder from the hopper to the die was set in the range of 180–200 °C, and the screw rotation speed was maintained at 100 rpm. The extruded strands were cooled and pelletized for subsequent processing.

### 2.4. Characterization and Properties

#### 2.4.1. FT-IR

The structural characteristics of the synthesized PP-g-IA were analyzed using Fourier-Transform Infrared Spectroscopy (FT-IR, Cary 630, Agilent Technologies, Santa Clara, CA, USA). The FT-IR spectra were recorded in the wavenumber range of 4000 to 600 cm^−1^.

#### 2.4.2. Scanning Electron Microscopy

The surface morphologies of the LC and mLC fibers, as well as the tensile fractured surfaces of the PP/lignocellulose composites, were observed using a FE-SEM (SU-8010, HITACHI, Tokyo, Japan). Prior to observation, all samples were sputter-coated with Platinum to prevent electron charging. The observations were performed at an accelerating voltage of 5 kV.

#### 2.4.3. Melt Flow Index

To evaluate the processability and melt viscosity of the PP/lignocellulose composites, the Melt Flow Index (MFI) was measured. MFI measurements were performed using a Melt Flow Indexer (WL-1400, With Lab, Anyang, Republic of Korea) according to the ISO 1133 standard [46]. The measurement conditions were set at a temperature of 230 °C and a load of 2.16 kg. The MFI value was calculated as the mass (g/10 min) of the composite extruded through the die over a 10 min period.

#### 2.4.4. Rheological Analysis

The rheological properties of the PP-based composites were evaluated using a strain-controlled rheometer (MCR 302e, Anton Paar, Graz, Austria) equipped with a parallel plate geometry (25 mm diameter). Oscillation frequency sweep tests were performed at a constant temperature of 190 °C over an angular frequency range from 100 to 0.1 rad/s. The strain amplitude was selected for each sample in order to fall within the linear viscoelastic region.

#### 2.4.5. Tensile Tests

The composite pellets were molded into ASTM D638 [47] Type I dumbbell-shaped specimens using an injection molding machine (WD-25, QNES Global, Incheon, Republic of Korea). During the injection molding process, the cylinder and mold temperatures were set at 200 °C and 110 °C, respectively. To evaluate the mechanical properties of the composites, tensile tests were conducted using a Universal Testing Machine (Instron 3400, Instron, Norwood, MA, USA) equipped with a 5 kN load cell. The testing speed was set at 5 mm/min, and all measurements were performed at room temperature (25 °C). An average value was calculated by testing replicate specimens for each composition. The tensile strength, elongation at break, and tensile modulus were determined.

#### 2.4.6. Thermogravimetric Analysis

To analyze the thermal stability and pyrolysis behavior of PP/lignocellulose composites, a thermogravimetric analyzer (TGA, TGA N-1000, Scinco, Seoul, Republic of Korea) was used. The analysis was conducted under a nitrogen atmosphere, heating from 30 °C to 600 °C at a heating rate of 10 °C/min declared.

#### 2.4.7. Statistical Analysis

Statistical analysis of the mechanical properties was performed using a one-way analysis of variance (ANOVA) in Microsoft Excel (Microsoft Corporation, Redmond, WA, USA). To ensure the validity of the measurements, 3 to 5 valid replicates were utilized for each composite formulation. All data are presented as the mean ± standard deviation (SD). Differences between the groups were considered statistically significant at a significance level of *p* < 0.05.

## 3. Results and Discussion

### 3.1. Synthesis and Characterization of PP-g-IA Compatibilizer

**Figure 1:**
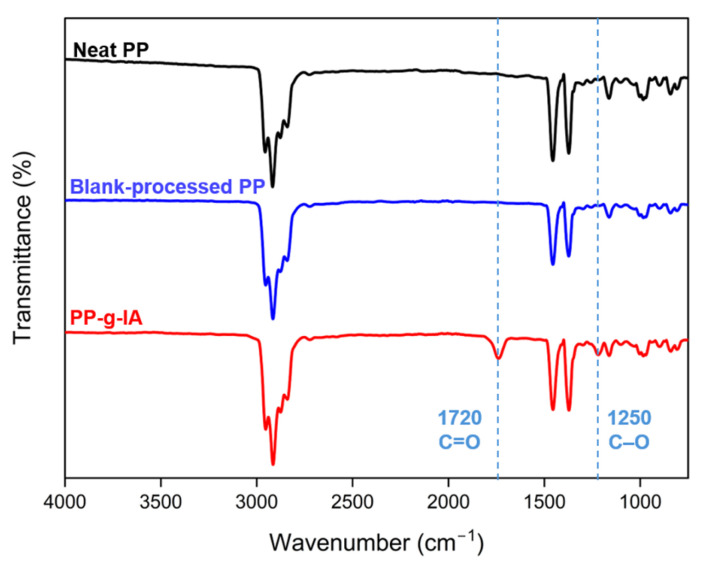
FT-IR spectra of neat PP, blank-processed PP and PP-g-IA.

FT-IR analysis was performed to verify the successful grafting of itaconic acid (IA) onto the polypropylene (PP) backbone. Figure 1 presents the FT-IR spectra of neat PP, blank-processed PP (processed at 190 °C for 7 min without additives), and the synthesized PP-g-IA. Both the neat PP and blank-processed PP spectra exhibited only the typical absorption bands of polypropylene, specifically C–H stretching vibrations (2800–2950 cm^−1^) and CH_2_ bending vibrations (1450 and 1376 cm^−1^), with no detectable peaks in the carbonyl region. This absence of carbonyl absorption in the blank-processed PP confirms that the thermo-mechanical processing conditions alone did not induce thermo-oxidative degradation. As reported by Shambilova et al. [35], thermo-oxidative degradation and subsequent carbonyl formation in PP typically require multiple reprocessing cycles; thus, the single-step processing in this study left the PP backbone intact against thermal oxidation.

In the PP-g-IA spectrum, however, two new and distinct absorption peaks emerged at 1720 cm^−1^ and 1250 cm^−1^ that were completely absent in the neat and blank-processed PP spectra. The peak at 1720 cm^−1^ is attributed to the C=O stretching vibration, while the peak at 1250 cm^−1^ corresponds to the C–O stretching vibration of the carboxylic acid groups (–COOH) originating from the grafted itaconic acid. Importantly, the simultaneous appearance of both the C=O and C–O stretching vibrations constitutes the characteristic chemical signature of carboxylic acid groups, a structural feature that cannot be formed by simple thermal oxidation of PP. Therefore, the combination of these two peaks provides definitive evidence that the IA monomers were successfully grafted onto the PP backbone rather than resulting from thermal degradation.

### 3.2. Lignocellulose Fiber Characterization

#### 3.2.1. Morphological Analysis of Lignocellulose

**Figure 2:**
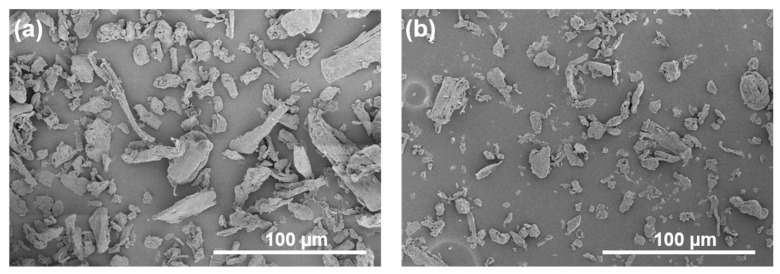
SEM images of lignocellulose: (**a**) LC and (**b**) mLC.

To clearly evaluate the morphological and dimensional changes induced by the surface modification, SEM observation was conducted. As shown in Figure 2, the mLC exhibits a noticeable reduction in both fiber length and diameter compared to the unmodified LC. This overall dimensional reduction is likely due to the PECVD processing conditions [45] inducing surface etching. To quantify this change, the dimensions of at least 100 individual fibers were measured using ImageJ software (version 1.54g, National Institutes of Health, Bethesda, MD, USA), strictly calibrating the pixel-to-length ratio using the 100 µm scale bar of each image to prevent scaling artifacts. The analysis revealed that the average aspect ratio (L/D) of the LC was 3.0 with a large standard deviation (±2.1), reflecting the inherently broad size distribution of raw natural fibers. In contrast, the mLC exhibited a significantly reduced aspect ratio of 2.1 with a much narrower standard deviation (±0.9) (Table 2). This reduction in both the average aspect ratio and its standard deviation indicates that the surface etching effectively broke down large fiber bundles, thereby narrowing the dimensional variation. This substantial decrease in the aspect ratio is anticipated to be a primary factor contributing to the reduction in the mechanical properties of the resulting composite materials.

#### 3.2.2. Surface Wettability of Lignocellulose

**Figure 3:**
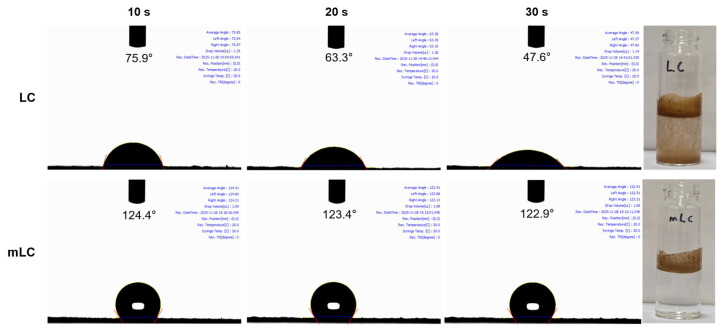
Changes in water contact angles of LC and mLC over time.

Following the morphological analysis, the water contact angle was measured to evaluate the changes in surface wettability of the LC before and after modification; the results are depicted in Figure 3.

As illustrated in Figure 3, the pristine LC exhibited an initial contact angle of 75.9° (at 10 s), which sharply declined to 47.6° after 30 s. This behavior is attributed to the inherent hydrophilicity arising from the abundance of hydroxyl (-OH) groups on the lignocellulosic surface. In contrast, the mLC showed an increased initial contact angle of 124.4°. Furthermore, it maintained a high contact angle of over 122.9° even after 30 s, demonstrating a stable state where water droplets did not penetrate the surface. These findings suggest that the surface energy was effectively reduced and hydrophobicity was successfully imparted through the plasma treatment.

To further confirm the contact angle results, a water dispersion test was performed. The LC powder dispersed relatively well in distilled water, turning the water cloudy and reflecting its inherent hydrophilicity. In contrast, the mLC powder floated on the water surface, exhibiting poor dispersibility and largely resisting wetting. This distinct difference in water dispersion clearly demonstrates that the mLC surface was successfully modified to be hydrophobic, which is expected to enhance its chemical compatibility with the nonpolar PP matrix.

### 3.3. Melt Flow Index (MFI) of Composites

**Figure 4:**
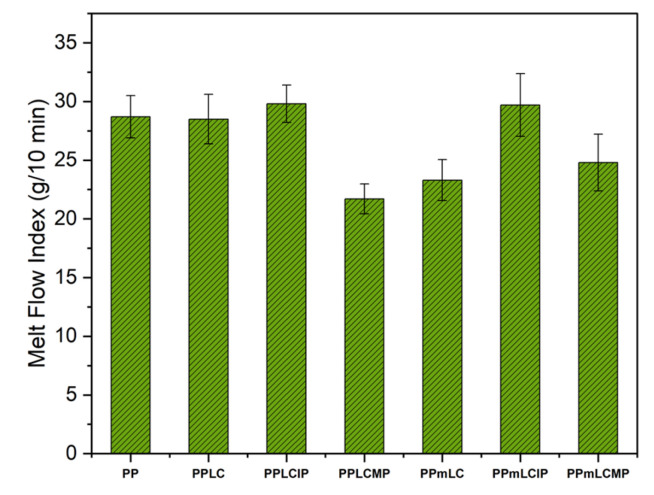
Melt flow index (MFI) of the samples: neat PP and PP/lignocellulose composites with different fibers and compatibilizers. (Error bars represent standard deviations).

To evaluate the processability and melt flow behavior of the composites, the melt flow index (MFI) was investigated. The MFI values of the PPLCIP and PPmLCIP samples were 29.8 g/10 min and 29.7 g/10 min (Figure 4), respectively, showing a slight increase compared to neat PP and LC composite samples without a compatibilizer. This increase in MFI can be interpreted as follows: the IP compatibilizer added to the composite is synthesized using an organic peroxide (DTBP) during its preparation. This behavior is consistent with the well-established phenomenon of peroxide-induced β-scission of PP chains during melt processing, which is known to reduce the average molecular weight and consequently increase the MFI [48,49]. Indeed, the PP-g-IA compatibilizer itself exhibits a remarkably high MFI value of 223.4 g/10 min (Table 1). Consequently, PPLCIP and PPmLCIP, which contain significantly lower-molecular-weight IP, show a reduced melt viscosity of the overall composite due to the influence of the low-molecular-weight components, resulting in relatively high MFI values. This is advantageous in terms of the processing flowability of the composite.

The PPLCMP sample exhibited the lowest MFI value among the measured samples (Figure 4). This is primarily attributed to the inherently higher molecular weight of the commercial PP-g-MAH (MP) compatibilizer, which undergoes comparatively less chain scission during its manufacturing process than the peroxide-initiated melt-grafted PP-g-IA (IP). As a result, the incorporation of MP contributes to a higher melt viscosity in the composite system. In contrast, the significantly higher MFI of PPLCIP reflects the extensive chain scission inherent to the synthesis of IP. In this regard, the contrasting MFI behaviors of PPLCIP and PPLCMP primarily reflect the difference in molecular weight between the two compatibilizers, rather than the degree of interfacial bonding.

### 3.4. Dynamic Rheological Characterization

**Figure 5:**
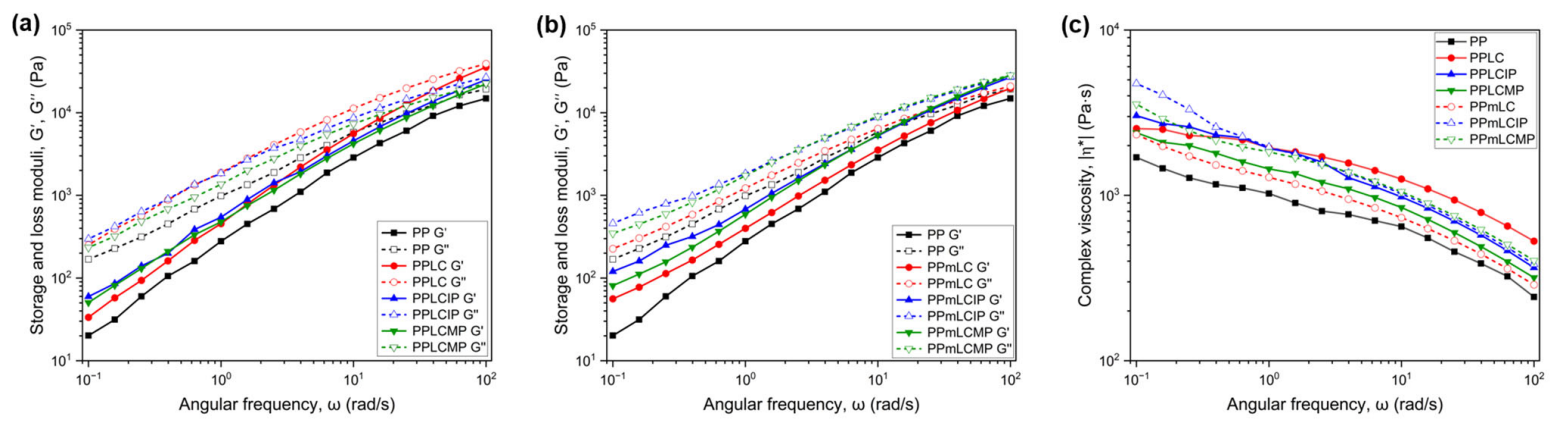
Dynamic rheological properties of the neat PP and PP/lignocellulose composites as a function of angular frequency (ω): (**a**) storage (G′) and loss (G″) moduli of the unmodified LC system, (**b**) storage (G′) and loss (G″) moduli of the modified mLC system, and (**c**) complex viscosity (|η*|) of all composite systems.

To elucidate the microstructural interactions and verify the compatibilization mechanisms of the composites, dynamic rheological behaviors—specifically the storage modulus (G′), loss modulus (G″), and complex viscosity (|η*|) as a function of angular frequency (ω)—were evaluated (Figure 5).

In the unmodified LC system, the uncompatibilized PPLC composite exhibited high G′ and G″ values in the high-frequency region (>10 rad/s) (Figure 5a). This is attributed to the presence of large fiber agglomerates caused by the severe polarity mismatch, which act as physical barriers and induce high internal friction during rapid shear flow. Upon the addition of the compatibilizers (both IP and MP), the high-frequency moduli and viscosity decreased due to the improved dispersion of agglomerates and the lubricating effect. Conversely, at the low-frequency terminal zone (0.1 rad/s), the G′ of both PPLCIP and PPLCMP surpassed that of PPLC. This pseudo-solid behavior indicates that the compatibilizers successfully restricted polymer chain relaxation by forming a strong chemical interfacial network through hydrogen bonding.

To evaluate the effect of fiber surface modification, the rheological behavior of the mLC system was investigated (Figure 5b). The uncompatibilized PPmLC exhibited the lowest moduli across the entire frequency range. While the plasma modification mitigated severe fiber agglomeration, the lack of chemical interfacial adhesion led to interfacial slippage between the nonpolar PP matrix and the fibers under shear stress. Upon the incorporation of either the IP or MP compatibilizer, the low-frequency moduli (PPmLCIP and PPmLCMP) significantly increased. Rather than robust chemical hydrogen bonding—which is limited by the depleted surface hydroxyl groups—this behavior in the melt state is driven by the hydrophobic nature and reduced length of the mLC fibers. These characteristics promote highly uniform fiber dispersion, establishing an extensive physical percolation network that heavily restricts melt flow.

The distinct network mechanisms are further corroborated by the complex viscosity behavior (Figure 5c). All compatibilized composites exhibited shear-thinning behavior, converging at high frequencies. At 0.1 rad/s, PPmLCIP showed the highest complex viscosity value overall. This maximum flow resistance is a direct physical manifestation of the dense morphological entanglement of the uniformly dispersed short fibers in the melt state, not an indicator of solid-state bond strength. Ultimately, these rheological responses provide strong, independent evidence that the bio-based PP-g-IA successfully establishes comprehensive networks—chemically driven in LC systems and physically driven in mLC systems—demonstrating rheological efficacy comparable to the conventional petroleum-based PP-g-MAH.

### 3.5. Mechanical Properties of Composites

**Figure 6:**
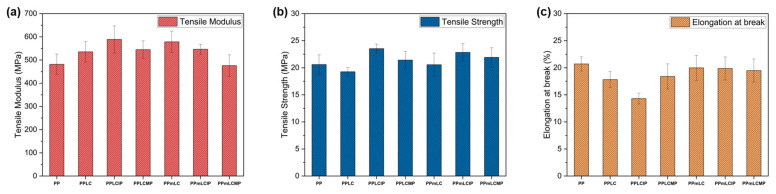
Mechanical properties of neat PP and PP/lignocellulose composites as a function of fiber modification and compatibilizer type: (**a**) tensile modulus, (**b**) tensile strength, and (**c**) elongation at break. (Error bars represent standard deviations).

**Figure 7:**
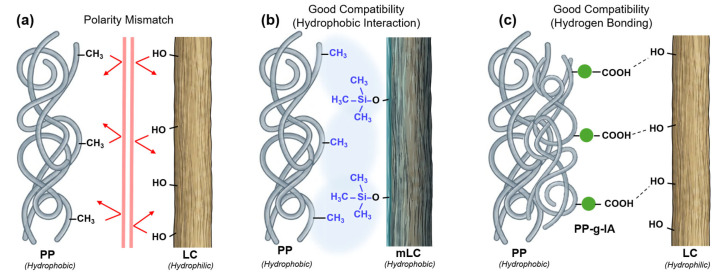
Schematic illustration of the interfacial compatibilization mechanisms: (**a**) polarity mismatch in the PP/LC, (**b**) hydrophobic interaction in the PP/mLC, and (**c**) hydrogen bonding facilitated by the bio-based PP-g-IA. The red arrows indicate the interfacial repulsion between the hydrophobic polypropylene (PP) matrix and the hydrophilic lignocellulose (LC) fibers. The blue chemical structures represent the surface modification of the fibers, and the green circles indicate the functional groups of the compatibilizer enhancing interfacial adhesion.

To systematically evaluate the reinforcing effects of the modified lignocellulose and the efficacy of the compatibilizers, the mechanical properties of the composites were investigated. As shown in Figure 6, without the addition of compatibilizers, the PPLC composite exhibited a slight decrease in tensile strength (19.3 MPa) compared to neat PP (20.6 MPa) due to poor interfacial adhesion between the highly non-polar PP matrix and the hydrophilic LC fibers (Figure 7a).

Interestingly, even without a compatibilizer, the PPmLC composite showed an improved tensile strength (20.6 MPa) and the highest Tensile modulus (578.7 MPa) among the uncompatibilized systems. This initial enhancement is primarily attributed to the chemical surface modification of the mLC induced by the plasma treatment. The treatment effectively reduces the inherent hydrophilicity of the LC fibers, thereby minimizing the surface energy difference with the highly non-polar PP matrix. Consequently, this improved interfacial wettability and compatibility allow the mLC composite to achieve higher strength despite its reduced aspect ratio.

However, upon the introduction of the compatibilizers, the trend changed significantly. The PPLCIP sample remarkably recorded the highest tensile strength (23.5 MPa) overall (*p* = 0.015). Although PPLCIP exhibited lower melt viscosity due to the incorporation of the low-molecular-weight PP-g-IA compatibilizer (as confirmed by the MFI analysis), which generally negatively impacts tensile strength [50,51], it successfully overcame this theoretical drawback. While a quantitative analysis of fiber dispersion (such as agglomerate size distribution) was not performed, the lowered matrix viscosity is highly expected to facilitate more uniform fiber dispersion during melt compounding. This proposed mechanism is qualitatively supported by the reduced high-frequency dynamic viscoelasticity (indicating less agglomerate-driven internal friction) and the highly cohesive, void-free fracture morphologies observed in SEM, which occurred alongside the robust interfacial bonding formed by the synthesized PP-g-IA (Figure 7c). Most importantly, once the interfacial adhesion issue was resolved by the IP compatibilizer, the inherently higher aspect ratio of the LC (compared to mLC) maximized the stress transfer efficiency, resulting in superior strength [1,20,21,22].

Furthermore, the compatibilizing efficiency of the synthesized PP-g-IA was found to be effective, demonstrating mechanical performance strictly comparable to the commercial PP-g-MAH (MP). For both fiber systems, the IP-compatibilized composites exhibited tensile strengths on par with their MP-compatibilized counterparts. It should be noted that the difference in molecular weight and chain architecture between the IP and MP makes it challenging to completely isolate the chemical compatibilization effect of IA from the rheological advantages of a lower viscosity matrix. Nevertheless, these results confirm that the synthesized bio-based PP-g-IA effectively acts as a reliable alternative, providing robust mechanical reliability comparable to established commercial compatibilizers.

While the PPLCIP composite exhibited a dramatic enhancement in tensile strength, the improvement in the PPmLCIP composite upon the addition of the IP compatibilizer was relatively marginal. This limited compatibilization effect in the mLC system is fundamentally attributed to its altered surface chemistry. Because the mLC was hydrophobically modified, its surface hydroxyl groups were significantly depleted. Consequently, the carboxylic acid groups of the PP-g-IA lacked sufficient reactive sites to form effective hydrogen bonds. Furthermore, although the PPmLCIP composite exhibited the highest low-frequency complex viscosity—driven by an extensive physical network of well-dispersed shorter fibers in the melt state—this physical entanglement alone is insufficient to withstand ultimate failure under extreme tensile stress. Ultimately, the lack of robust hydrogen bonding, compounded by the inherently lower aspect ratio, elucidates why the PPmLCIP system could not achieve the efficient macroscopic stress transfer observed in the PPLCIP system.

### 3.6. Morphology Analysis of Composites

**Figure 8:**
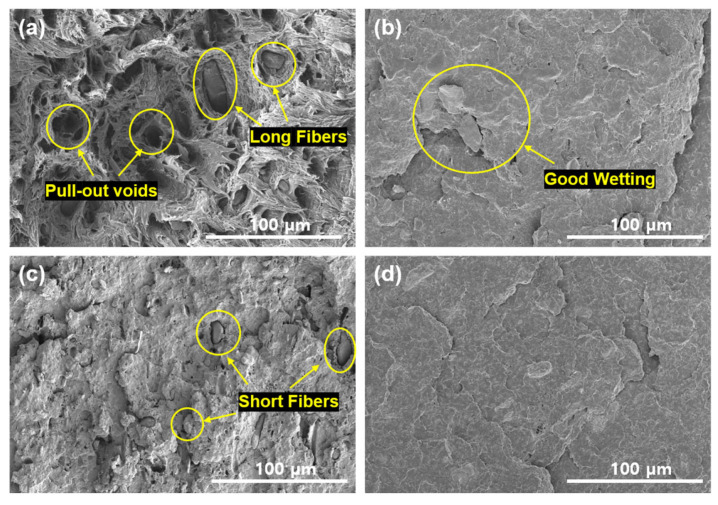
SEM images of PP/Lignocellulose composite fracture surfaces: (**a**) PPLC, (**b**) PPLCIP, (**c**) PPmLC, (**d**) PPmLCIP.

To provide morphological evidence for the interfacial interactions between the PP matrix and the lignocellulose fibers, the tensile fracture surfaces were observed via SEM (Figure 8). Without surface modification or compatibilization, the fracture surface of the PPLC composite is dominated by large interfacial voids with a distinct lack of PP matrix surrounding the exposed fibers, despite the higher aspect ratio of the LC (Figure 8a). This indicates a severe lack of interfacial adhesion due to the inherent polarity mismatch between the nonpolar PP and polar LC, leaving the higher fibers completely debonded from the matrix without any close contact. This morphological evidence indicates that the applied tensile stress could not be effectively transferred across the interface, leading to suboptimal reinforcement.

Conversely, the PPmLC composite exhibits a noticeably different interfacial morphology characterized by close proximity and tight wrapping of the PP matrix around the mLC fibers (Figure 8c). This minimized interfacial gap demonstrates that the hydrophobic modification enhanced the chemical compatibility and affinity between the mLC and the PP matrix. Consequently, despite the physical disadvantage of a lower fiber length, this closer matrix–fiber interaction facilitated more effective stress transfer, directly supporting the higher tensile strength observed in the PPmLC composite compared to the PPLC.

Furthermore, the incorporation of the IP compatibilizer induced a morphological transformation toward a highly cohesive and dense texture with no obvious fiber pull-out voids for both composite systems (Figure 8b,d). Particularly in the PPLCIP composite, the LC fibers are seamlessly embedded and tightly coated by the PP matrix (highlighted as ‘Good Wetting’ in Figure 8b), confirming robust interfacial adhesion. The IP compatibilizer effectively bridged the PP matrix and the LC surface, maximizing interfacial stress transfer. Consequently, the synergistic combination of the preserved higher aspect ratio of LC and the tight interfacial wetting provided by IP perfectly corroborates why the PPLCIP composite achieved the highest tensile strength in this study.

### 3.7. Thermal Stability of Composites

**Figure 9:**
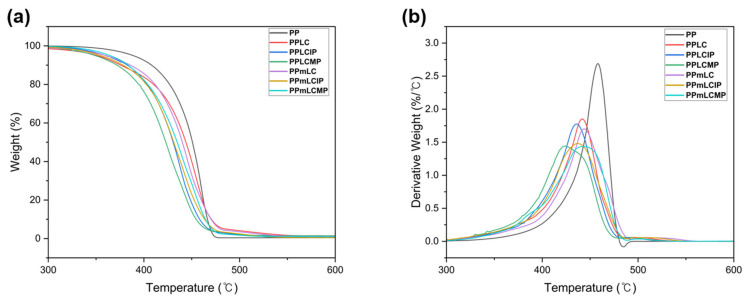
TGA and DTG curves of PP/Lignocellulose composites: (**a**) TGA curves and (**b**) DTG curves.

The thermal stability and degradation behavior of the neat PP and composites were evaluated using TGA and DTG. The corresponding curves are presented in Figure 9, and the specific degradation temperatures are summarized in Table 3.

The neat PP exhibited the highest initial degradation temperature (T_d5%_) of 388.2 °C and maximum degradation temperature (T_max_) of 458.9 °C. Upon the incorporation of LC and mLC fibers, the overall thermal stability of the composites decreased. This initial reduction is primarily due to the inherently lower thermal decomposition temperature of the lignocellulosic components (hemicellulose and cellulose) compared to the highly stable aliphatic carbon backbone of PP.

The early thermal degradation behavior (T_d5%_) showed a general decrease upon the incorporation of compatibilizers, which may initially appear counterintuitive given the improved interfacial adhesion. However, this reduction is primarily attributed to the inherent thermal vulnerability of the compatibilizers themselves. Both IP and MP are composed of relatively low-molecular-weight polymer chains bearing highly reactive functional groups: carboxylic acid for IP and maleic anhydride for MP. At the early stage of thermal decomposition, these thermally sensitive functional groups and the lower-molecular-weight compatibilizer backbone readily undergo initial cleavage, acting as the weakest thermal links within the composite matrix. Consequently, the compatibilizer introduces preferential early degradation pathways that can offset any thermal barrier effect provided by improved fiber–matrix interfacial adhesion, leading to a decrease or limited improvement in T_d5%_.

Furthermore, at elevated temperatures, the addition of the compatibilizers (IP and MP) led to a general decrease in T_max_ for both the LC and mLC systems. For instance, the T_max_ of PPmLC (446.0 °C) dropped to 440.3 °C (PPmLCIP) and 438.5 °C (PPmLCMP) upon compatibilization. This overall reduction in peak thermal stability is a direct consequence of the intrinsic thermal vulnerability of the compatibilizers themselves. Both the synthesized IP and the commercial MP possess highly reactive, thermally sensitive functional groups: carboxylic acid for IP and maleic anhydride for MP. At elevated temperatures, these functional groups readily undergo early thermal cleavage, such as dehydration or decarboxylation. Consequently, these reactive groups initiate thermal degradation at lower temperatures and accelerate the overall mass loss process, which physically overrides any potential thermal barrier effect at the peak degradation stage.

## 4. Conclusions

This study synthesized a bio-based PP-g-IA compatibilizer and comprehensively analyzed complex compatibilization strategies involving LC surface modification and compatibilizer types. In the uncompatibilized systems, the mLC secured better chemical compatibility with the nonpolar PP matrix, exhibiting higher tensile strength than the PPLC despite its reduced aspect ratio. However, upon the incorporation of the PP-g-IA compatibilizer, the interfacial bonding issue between the polar LC and nonpolar PP was significantly improved. Dynamic rheological analysis confirmed that PP-g-IA effectively suppressed severe fiber agglomeration and established a robust 3-dimensional interfacial network. Consequently, the physical reinforcement effect of LC’s higher aspect ratio became the dominant factor, allowing the PPLCIP composite to achieve the highest tensile strength (23.5 MPa) in this study through the synergy of a higher aspect ratio and excellent interfacial adhesion.

Furthermore, the composite compatibilized by the synthesized bio-based PP-g-IA demonstrated tensile strength comparable to that with the conventional petroleum-based PP-g-MAH, while simultaneously providing enhanced melt flowability. In conclusion, this study clearly elucidates the interactive mechanism between chemical compatibility and physical aspect ratio, demonstrating that the bio-based PP-g-IA is an efficient and eco-friendly compatibilization strategy. It can serve as a viable and sustainable alternative to existing petroleum-based compatibilizers, providing comparable mechanical reliability for eco-friendly biocomposites.

## Figures and Tables

**Table 1:** PP/Lignocellulose composite compositions.

	PP (wt.%)	LC ^a^ (wt.%)	mLC ^b^ (wt.%)	IP ^c^ (phr ^e^)	MP ^d^ (phr)
PPLC	90	10	0	0	0
PPLCIP	90	10	0	5	0
PPLCMP	90	10	0	0	5
PPmLC	90	0	10	0	0
PPmLCIP	90	0	10	5	0
PPmLCMP	90	0	10	0	5

LC ^a^: Lignocellulose; mLC ^b^: Hydrophobically Modified Lignocellulose; IP ^c^: Itaconic Acid-grafted Polypropylene (PP-g-IA), MFI: 223.4 g/10 min; MP ^d^: Maleic Anhydride-grafted Polypropylene (PP-g-MAH), MFI: 78.5 g/10 min; phr ^e^: parts per hundred resin.

**Table 2:** Fiber size of LC and mLC. (Data are presented as mean ± standard deviation).

Fiber	Diameter (µm)	Length (µm)	Aspect Ratio (l/d)
LC	7.6 ± 3.8	20.4 ± 13.7	3.0 ± 2.1
mLC	5.3 ± 2.8	10.8 ± 6.1	2.1 ± 0.9

**Table 3:** Thermal Decomposition Temperature of PP/Lignocellulose composites determined by TGA.

NAME	T_d5%_ (°C)	T_d50%_ (°C)	T_d95%_ (°C)	T_max_ (°C)
PP	388.2	450.7	469.4	458.9
PPLC	356.2	435.0	472.8	444.7
PPLCIP	359.9	430.6	467.5	438.2
PPLCMP	348.8	422.8	465.1	424.3
PPmLC	359.9	440.3	480.0	446.0
PPmLCIP	354.7	431.1	473.0	440.3
PPmLCMP	364.6	434.8	471.1	438.5

## Data Availability

The original contributions presented in this study are included in the article. Further inquiries can be directed to the corresponding author.

## Abbreviations

The following abbreviations are used in this manuscript:

PP	Polypropylene
IA	Itaconic acid
PP-g-IA(IP)	Itaconic acid-grafted polypropylene
MAH	Maleic anhydride
PP-g-MAH(MP)	Maleic anhydride-grafted polypropylene
LC	Lignocellulose
mLC	Hydrophobically modified lignocellulose
MFI	Melt Flow Index
UTM	Universal Testing Machine
SEM	Scanning Electron Microscope
TGA	Thermogravimetric Analysis
